# Mr. Wharton Jones's Researches in Embryology

**Published:** 1840-01

**Authors:** 


### MR. WHARTON JONES'S RESEARCHES IN EMBRYOLOGY.

We owe it to our readers, and especially to Mr. Wharton Jones, to correct an error in the first Article of our present Number, which was not discovered until too late to be altered in the proper place. It is at page 6, line 7 from the bottom; and we request the reader to insert in that line, "man," or "the human ovum," in place of "the rabbit." The paragraph was intended to present, in a compressed form, the successive steps of discovery by which it has been established, that the germ-vesicle prevails generally throughout the animal series as an element of the ovarian ovum. And Mr. Jones's discovery of that vesicle in the human ovum (presumed by him at the time to be, with his other observations of the same in the pig, rabbit, &c., the earliest evidence of its existence in the class mammalia,) is one of those important steps. With respect to the germ-spot, Mr. Jones, placing greater reliance on his own observations, made with the simple microscope, than on those of others where the compressor was used, adheres, as we understand, to his first description of it, and maintains it to be an elevation on the side of the vesicle. We beg to add, that, in Mr. Jones's valuable paper, the same resemblance between the mature ovarian ovum of a mammal and the immature ovarian ovum of a bird is insisted on, which we have presented at greater length from Valentin, at page 9. In his second paper, besides those just views, as we conceive them, respecting the origin of the chorion, which we have noticed in the body of our article, Mr. Jones also details his observations and inferences from them relative to the genesis of the blastoderma: a subject of which the interest is equalled by the delicacy and the difficulty of the research. This subject must soon engage the attention of other observers more directly than it has hitherto done, when these original observations will demand a special discussion. We allude to them now for the purpose of recording, that here is detailed the dissection of a human ovum, probably, as Dr. A. Thomson remarks, "the very earliest that has been accurately described." "The whole cavity of the chorion," says Mr. Jones, "was filled with a fine gelatinous cellular tissue, imbedded in which, towards one extremity of the ovum, was a small round body. It was evidently the spherical blastoderma; on being taken out, and examined under the microscope, it presented the same friable globular structure found in the spherical blastoderma of the rabbit. . . . . There was no vitellary membrane to be seen"

